# Primary pulmonary artery sarcoma masquerading as pulmonary thromboembolism: a rare diagnosis unveiled

**DOI:** 10.1186/s13569-017-0080-8

**Published:** 2017-07-01

**Authors:** Abhishek Mahajan, Bharat Rekhi, Siddhartha Laskar, Jyoti Bajpai, Lekshmy Jayasree, Meenakshi H. Thakur

**Affiliations:** 1 Department of Radiodiagnosis and Imaging, Tata Memorial Hospital, Tata Memorial Centre, Room No 120, Dr E Borges Road, Parel, Mumbai, Maharashtra 400012 India; 2 Department of Pathology, Tata Memorial Hospital, Tata Memorial Centre, Mumbai, Maharashtra 400012 India; 3 Department of Radiation Oncology, Tata Memorial Hospital, Tata Memorial Centre, Mumbai, Maharashtra 400012 India; 4 Department of Medical Oncology, Tata Memorial Hospital, Tata Memorial Centre, Mumbai, 400012 India

**Keywords:** Pulmonary artery, Sarcoma, Vascular neoplasms, MRI, CT, Cardiac MRI, Immunohistochemistry

## Abstract

**Background:**

Primary pulmonary artery sarcomas are rare malignant vascular tumors and carry a very poor prognosis. Due to overlapping clinical and radiological features, the differentiation between pulmonary artery thromboembolism and pulmonary artery sarcoma can be challenging.

**Case presentation:**

We herein present clinical, radiological and pathological features of primary pulmonary artery high grade sarcoma (angiosarcoma) in a 59-year-old male. The patient presented with a history of breathlessness on exertion of 2-months duration and was misdiagnosed as massive pulmonary thromboembolism on initial CT imaging.

**Conclusion:**

Great similarity with significant degree of overlap in clinical and radiologic presentation makes differentiation of pulmonary artery sarcomas and thromboembolism a diagnostic challenge. Even though they are exceptionally rare, one should always consider it as differential diagnosis especially in cases with atypical clinical or imaging presentation.

## Background

Primary angiosarcomas are rare malignant tumors of epithelial origin and account for 2% of the all sarcomas. A few reports have described primary angiosarcomas in the anterior mediastinum, typically without an obvious vascular origin. Intrapulmonary angiosarcomas are even rarer and only most of the reported cases have been identified on autopsy [1].

## Case

**Fig. 1:**
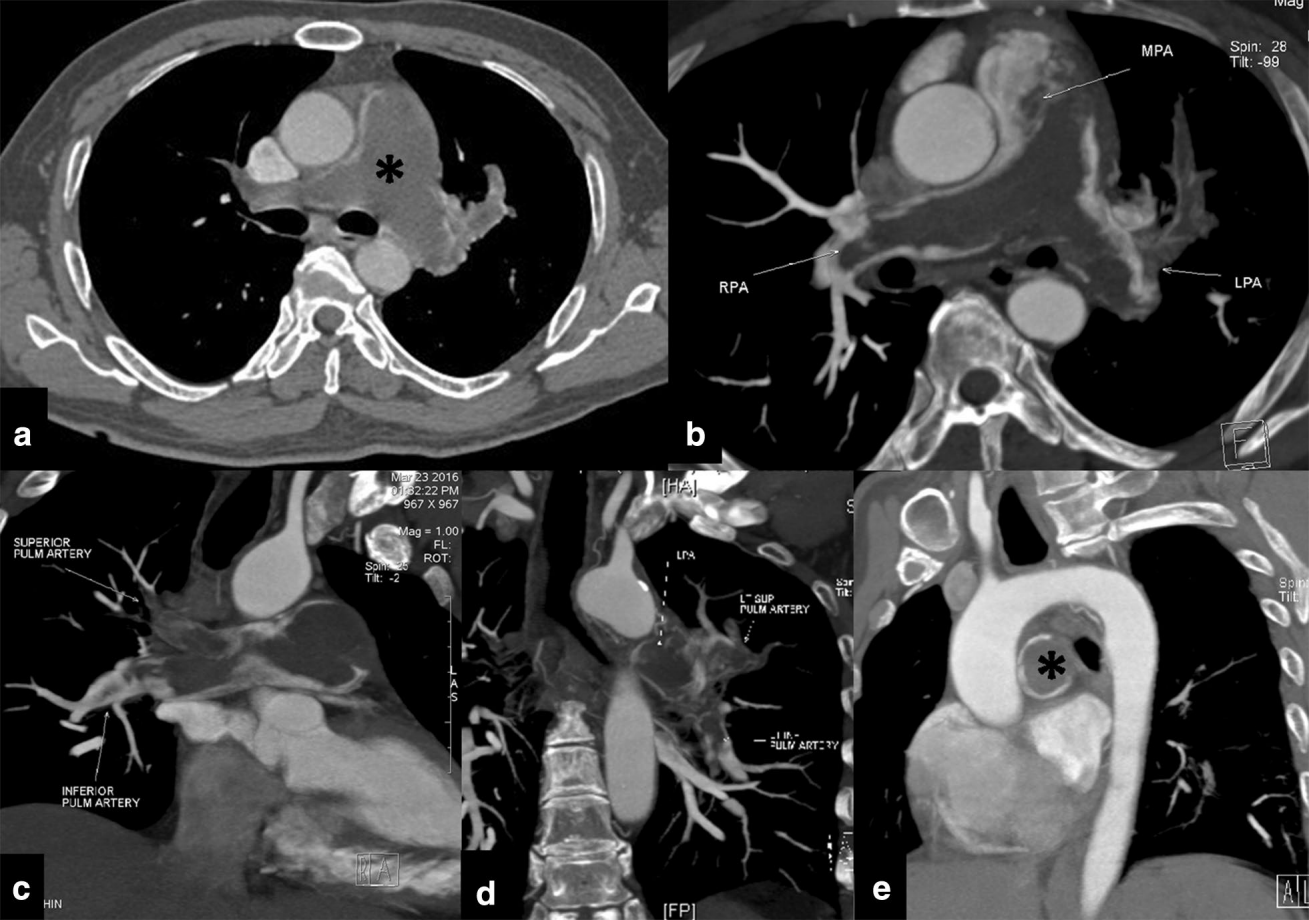
Contrast enhanced CT imaging of the thorax (**a**, **b** axial; **c**, **d** oblique coronal; **e** sagittal reformatted images) shows a hypodense filling defect (*asterisk*) in the main and bilateral pulmonary arteries which does not exhibit post contrast enhancement (*MPA* main pulmonary artery, *RPA* right pulmonary artery, *LPA* left pulmonary artery). No pulmonary parenchymal abnormality was noted

**Fig. 2:**
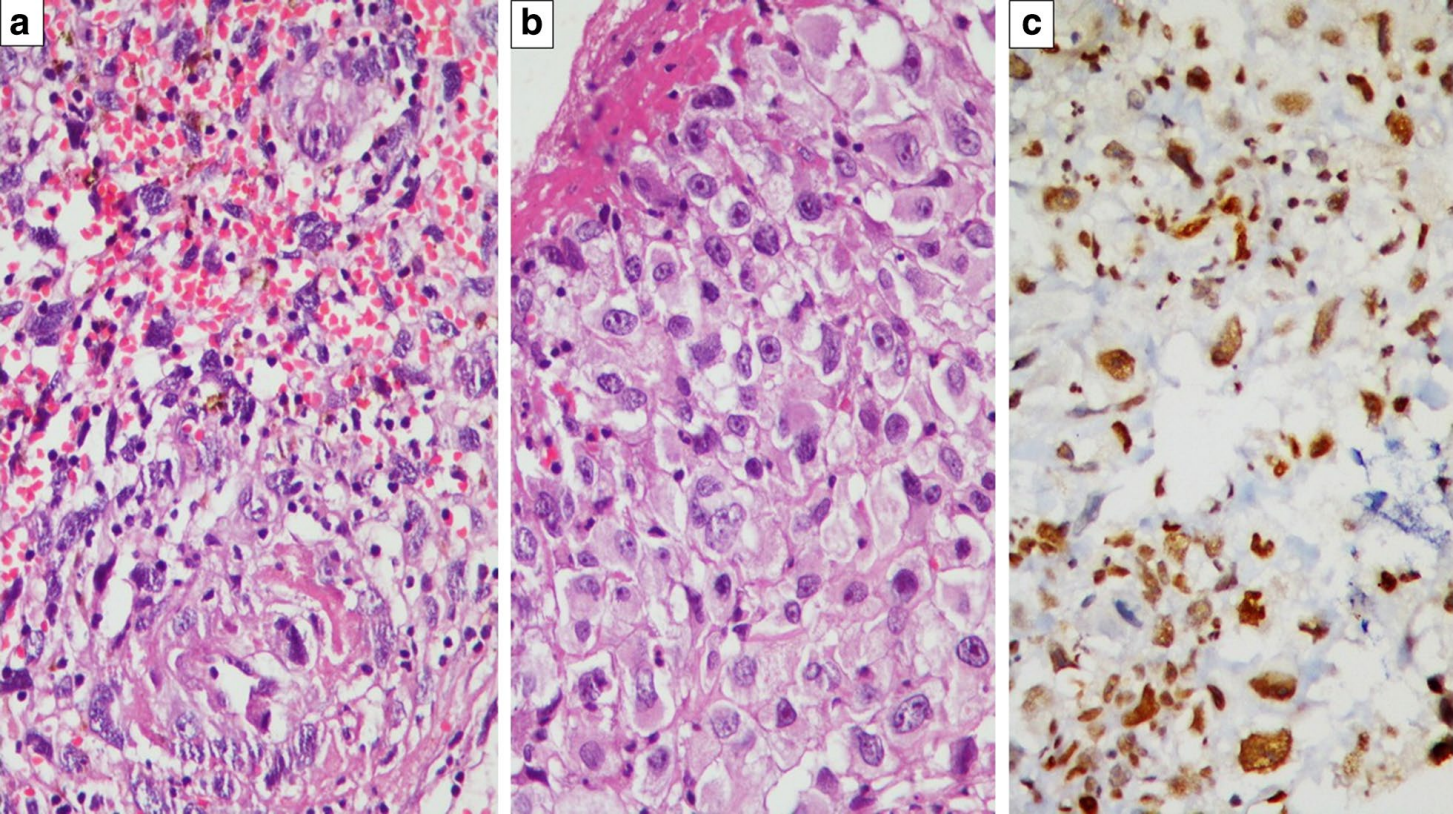
**a** Sarcomatous tumor with intratumoral haemorrhage and fibrinoid necrosis [Haematoxylin and Eosin (H and E) stain ×200]. **b** Higher magnification showing tumor cells with focal epithelioid morphology, exhibiting moderate to marked nuclear pleomorphism [H and E ×400]. **c** By Immunohistochemistry, tumor cells showing diffuse intranuclear Fli1 positivity (diaminobenzidine immunostain ×400)

A 59-year-old male, known diabetic and hypertensive for the past 5 years and on medications, presented with a history of breathlessness on exertion for the past 2 months. There was no history of chest pain, cough, palpitations or syncope. On examination no significant findings could be elicited. Laboratory investigations and complete haematological profile was within normal limits (no features of hypercoagulable disorders). Echocardiography showed dilated right atrium and right ventricle with impaired right ventricular systolic function. The pulmonary valve appeared thickened with possible mass attached to it, with turbulence and gradient in the main pulmonary artery. A contrast enhanced CT was performed which revealed a hypodense filling defect in the main and bilateral pulmonary arteries which did not exhibit any significant post contrast enhancement (Fig. 1). Based on the clinical and radiological findings a diagnosis of pulmonary thromboembolism was made and pulmonary thromboendarterectomy was planned. Under GA, midline sternotomy was performed and main pulmonary artery was opened. Grey white organised mass with thrombosed areas was removed by separating it from the main pulmonary artery, bilateral pulmonary artery branches and pulmonary valves. On histopathology the tumor was composed of spindle to epithelioid cells exhibiting moderate to marked pleomorphism with vascular growth pattern and prominent areas of intratumoral haemorrhage and fibrinoid necrosis. Interspersed were mitotic figures and areas of focal myxoid change showed features of high grade sarcoma involving the media and intima and the lumen of the pulmonary artery with epithelioid morphology (Fig. 2a, b). On Immunohistochemistry, tumor cells were diffusely positive for Fli1 (Fig. 2c), which is a surrogate vascular marker; focally weakly positive for CD31, while were negative for CD34. Tumor cells were negative for SMA, desmin and H-caldesmon (myogenic/myofibroblastic markers). The final diagnosis was primary pulmonary artery high grade sarcoma and the suggestive histogenesis was vascular (angiosarcoma).

**Fig. 3:**
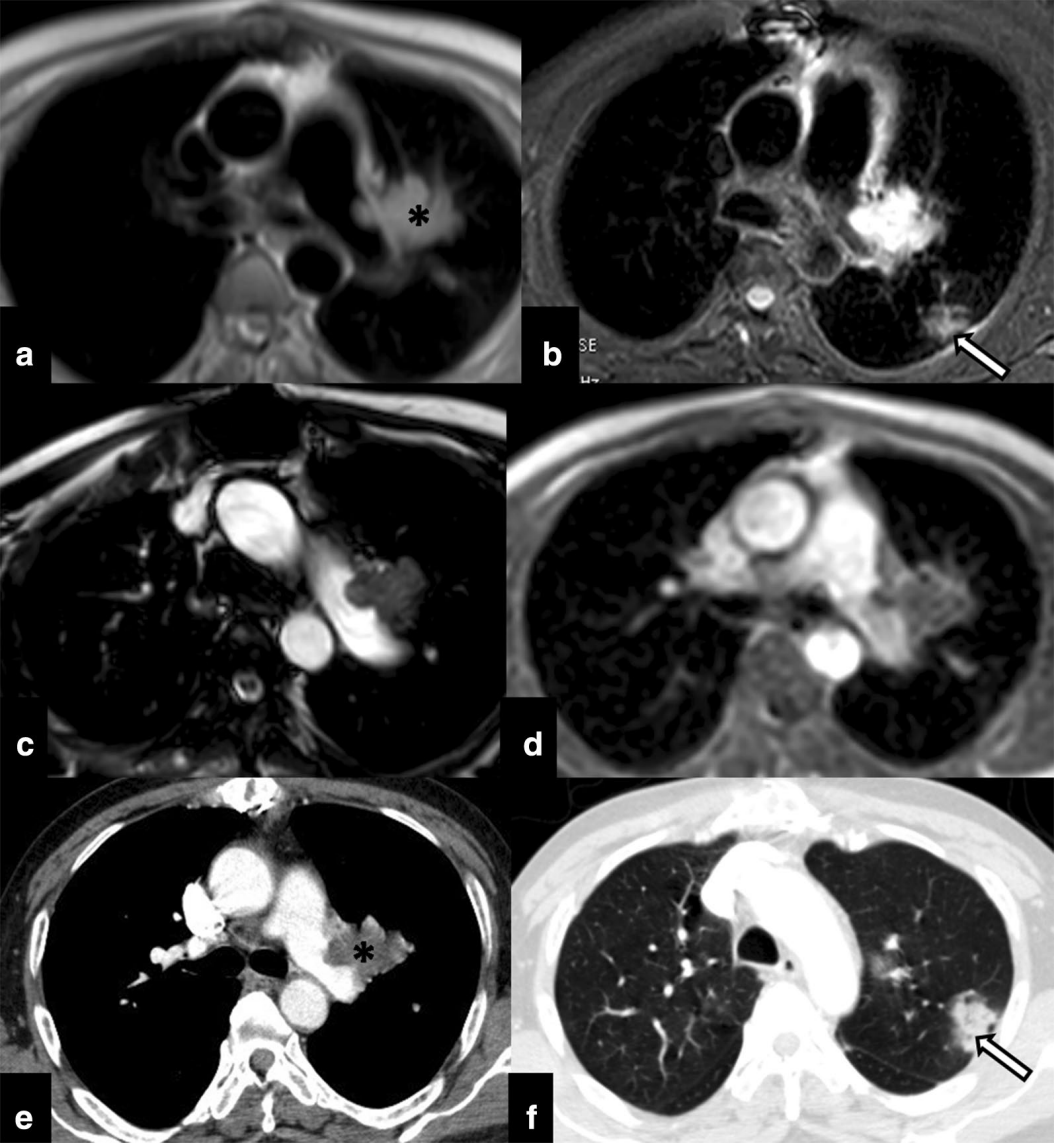
Post pulmonary endarterectomy cine-cardiac MRI (axial **a**, **b**, **c**, **d** T1W; **b** STIR, T2 and Post-contrast respectively) shows residual disease (*asterisk*) in the left pulmonary trunk with exophytic extravascular component. Focal patchy consolidation like opacities (*arrow*) were also seen in the lung parenchyma which were in favour of chronic thromboembolic phenomenon related infarcts presenting as consolidation (confirmed on post-contrast CT thorax: **e** mediastinal window and **f** lung window)

**Fig. 4:**
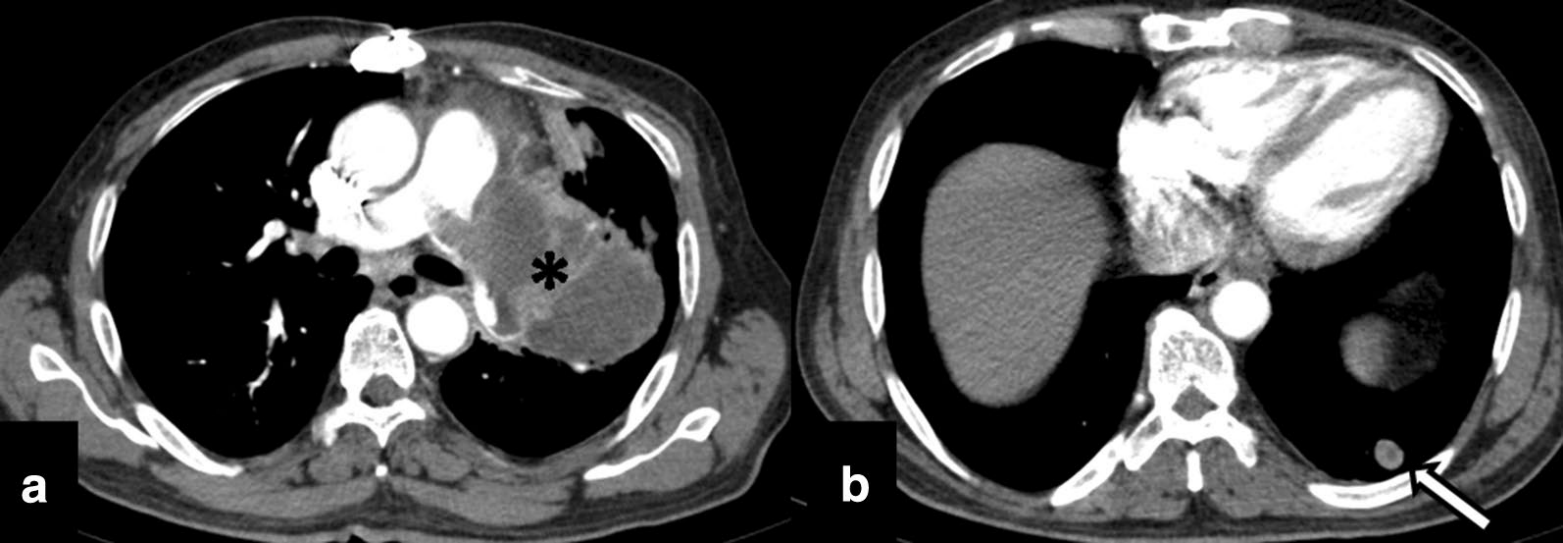
Follow-up imaging after 12 cycles of radiotherapy shows a large lobulated peripherally enhancing exophytic mediastinal mass (*asterisk*) arising from the left main pulmonary artery and invading its segmental arteries with a well-defined soft tissue nodule in left lower (*arrow*). CT findings were suggestive of disease progression with pulmonary parenchymal metastasis

The immediate post-operative period was uneventful. Two-month post-surgery patient present with on and off pleuritic type of chest pain and was referred to our tertiary care centre for further management. As a part of the work up, Cine-cardiac MRI was performed which showed non-enhancing altered signal intensity lobulated mass in the left pulmonary artery with both exophytic and endophytic intravascular component (Fig. 3a–d). CT was performed to rule out pulmonary metastatic disease however CT thorax revealed ill-defined focal patchy pneumonic areas in bilateral lung parenchyma that were in favour of pulmonary changes as a sequel to chronic thromboembolic phenomenon (Fig. 3e, f). In view of the progressive residual disease patient was treated with definitive external beam radiotherapy (60 Gy in 30 fractions; dose per fraction: 200 cGy). During the course of treatment patient had occasional haemoptysis which resolved spontaneously. Follow-up imaging after 12 cycles of radiotherapy revealed significant progression of the primary disease with new development of pulmonary parenchymal metastasis (Fig. 4). In view of the disease progression with short disease free interval period the patient is currently treated with metronomic therapy.

## Discussion

Primary pulmonary angiosarcomas are asymptomatic or manifest with varied presentation as massive pulmonary haemorrhage or haemoptysis, spontaneous haemothorax, multiple bilateral nodules on chest radiograph, pleuritic chest pain, non-productive cough or dyspnoea. These tumors usually occur in middle-aged adults, and the clinical symptoms depend on their location. These tumors have been associated with radiation therapy and with occupational exposure to vinyl chloride and arsenic [2, 3].

At radiography, extracardiac mediastinal sarcomas manifest as large masses. At CT and MR imaging, cardiac angiosarcomas manifest as diffuse wall thickening or focal masses that typically involve the right atrium and enhance heterogeneously after intravenous administration of contrast material. Primary pulmonary angiosarcomas usually manifest as multiple bilateral nodules. The attenuation at CT can be homogeneous but is more often heterogeneous due to areas of haemorrhage, necrosis, and cyst formation. MRI scores over CT in detecting the subtle post-contrast heterogeneity that helps in ruling out bland pulmonary artery thrombosis and also provides more accurate delineation of the extent of local disease than CT [2, 3]. The degree of contrast enhancement on MR imaging has shown correlation with the degree of tumor differentiation, content of myxoid matrix, and associated thrombus. Post-therapy changes in tumor size and morphology such as identification of areas of intratumoral necrosis or haemorrhage, makes gadolinium-enhanced MRI an important modality for response assessment [3, 4]. Other invasive imaging modalities used in difficult cases include are pulmonary artery angiography and intravascular ultrasound. On angiography pulmonary artery sarcomas may be seen as polypoid filling defect that moves with the cardiac cycle. Angiography can also be used to measure pressure gradients across the mass. Intravascular ultrasound is helpful in determining the extent of pulmonary valve involvement [3, 4].

Thromboembolic disease of the pulmonary artery is a close clinical and radiological mimic of this rare condition and almost always poses an as challenging dogma. Though the reported incidence of primary pulmonary artery angiosarcoma is very low it should always be considered a potential differential diagnosis for pulmonary thromboembolism, especially in scenarios where there is no identifiable cause thromboembolic event or the patient is not responding to anticoagulant therapy [4, 5]. PET CT is helpful in differentiating them and sarcomas are metabolically active (FDG positive) and thrombus is bland (FDG negative). FDG-PET/CT is also advised to rule out metastases. Other rare differential diagnosis includes (1) carcinomatous arteriopathy (pulmonary tumor thrombotic microangiopathy), (2) benign vasoformative lesions such as pulmonary capillary hemangiomatosis and diffuse pulmonary lymphangiomatosis, (3) primary intrathoracic malignancies with intravascular or perivascular invasion and metastasis, (4) alveolar haemorrhage syndromes, (5) Kaposis sarcoma, (6) pseudovascular sarcomatoid carcinoma, (7) metastatic angiosarcoma [4, 5].

On histopathology, macroscopically angiosarcomas are characterised by a large mass of tan coloured fleshy tissue, often with central necrosis and haemorrhage [6]. Microscopically there are clusters of round to oval/polygonal epithelial cells with erythrophagocytosis. The histopathological subclassfication of pulmonary artery sarcoma includes undifferentiated rhabdomyosarcoma, angiosarcoma, fibrosarcoma, malignant mesenchymoma, myxosarcoma, chondrosarcoma, osteosarcoma, malignant fibrous histiocytoma, unclassified leiomyosarcomas [6, 7]. Using IHC antibody markers of endothelial differentiation help diagnosing vasoformative nature of tumor and differentiate them from other histologic mimics. These commonly used antibodies include antibodies directed against Fli1, CD-31 glycoprotein, PECAM- (platelet endothelium cell adhesion molecule), von Willebrand factor (Factor VIII-related antigen) and CD-34 protein (human hematopoietic progenitor cell antigen [7, 8].

Even after adequate resection most of these patients have a very poor quality of life. The prognosis is poor, with few patients surviving beyond 3 years with multimodality treatment (survival of 24.7 ± 8.5 months for patients treated with multimodality treatment versus 8.0 ± 1.7 months for patients treated with single-modality) [2, 3]. Literature suggests that radical surgery with wide resection margins is the treatment of choice for patients with localised disease however as most cases present in later stage and surgical resection offers palliation. Recent studies have shown benefit from neoadjuvant or adjuvant radiotherapy, chemotherapy or both [3]. Chemotherapy has been used to decrease the size of the tumor prior to surgery to facilitate resection, as well as in the treatment of metastatic disease [2, 3].

To conclude, great similarity with significant degree of overlap in clinical and radiologic presentation makes differentiation of pulmonary artery sarcomas and thromboembolism a diagnostic challenge. Even though they are exceptionally rare, one should always consider it as differential diagnosis especially in cases with atypical clinical or imaging presentation. Cine Cardiac MRI as adjunct to CT imaging increases the diagnostic accuracy and helps in timely diagnosis and appropriate management.
